# Synthetic spatial patterning in bacteria: advances based on novel diffusible signals

**DOI:** 10.1111/1751-7915.13979

**Published:** 2021-11-29

**Authors:** Martina Oliver Huidobro, Jure Tica, Georg K. A. Wachter, Mark Isalan

**Affiliations:** ^1^ Department of Life Sciences Imperial College London London SW7 2AZ UK

## Abstract

Engineering multicellular patterning may help in the understanding of some fundamental laws of pattern formation and thus may contribute to the field of developmental biology. Furthermore, advanced spatial control over gene expression may revolutionize fields such as medicine, through organoid or tissue engineering. To date, foundational advances in spatial synthetic biology have often been made in prokaryotes, using artificial gene circuits. In this review, engineered patterns are classified into four levels of increasing complexity, ranging from spatial systems with no diffusible signals to systems with complex multi‐diffusor interactions. This classification highlights how the field was held back by a lack of diffusible components. Consequently, we provide a summary of both previously characterized and some new potential candidate small‐molecule signals that can regulate gene expression in *Escherichia coli*. These diffusive signals will help synthetic biologists to successfully engineer increasingly intricate, robust and tuneable spatial structures.

## Graphical abstract

Engineering multicellular patterning may help in the understanding of some fundamental laws of pattern formation. In this review, engineered patterns are classified into four levels of increasing complexity, ranging from spatial systems with no diffusible signals to systems with complex multi‐diffusor interactions. We provide a summary of candidate small molecule signals to help synthetic biologists to engineer tuneable spatial structures.

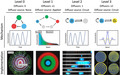

## Introduction

Biological patterning can be defined as the organized arrangement of an organism’s features (Davies, 2017), where an initially uniform field of cells gains complexity and heterogeneity in the spatial domain (Murray, 2013; Davies and Glykofrydis, 2020). This structure is crucial for function in multicellular organisms (Blest, 1957; Stevens *et al*., 2006; Strauss *et al*., 2020).

Understanding the mechanisms behind patterning is difficult due to the tangled nature of biology. The study of developmental biology largely consists in observing embryos or tissues and in perturbing the systems to validate different hypotheses (Murray *et al*., 2011; Raspopovic *et al*., 2014). This approach leads to insights into the complexity of a particular biological system. In contrast, in synthetic biology a system is built from first principles, making it simpler, more controllable and insulated from the natural genetic context (Nielsen *et al*., 2016; Meyer *et al*., 2019). Building the desired patterns with a synthetic system is one step towards showing that these basic principles can potentially occur in biology (Davies, 2017; Luo *et al*., 2019; Santos‐Moreno and Schaerli, 2019). However, it is important to acknowledge that successful engineering does not necessarily imply the occurrence of specific mechanisms in natural systems. Synthetic patterning systems nonetheless provide powerful tools for bioengineering and offer a proof that these mechanisms could potentially occur in development.

In addition to expanding our knowledge of developmental biology, building a simple and programmable system is necessary for the synthesis of patterned tissues, organoids or biofilms for downstream biotechnology applications (Scholes and Isalan, 2017; Davies and Glykofrydis, 2020). To work towards this goal, bacteria provide a relatively simple chassis, where spatial systems can be built in a controlled manner from first principles, using modelling to guide engineering (Elowitz and Leibler, 2000; Gardner *et al*., 2000; Salis *et al*., 2009).

This review outlines the progress in synthetic patterning using *Escherichia coli* and focuses on how the field was historically held back by a lack of diffusible components. It then highlights recently characterized components that can be used to build more complex, multi‐diffusor systems. While interesting patterning systems were also engineered in other cellular systems (Cachat *et al*., 2017; Sekine *et al*., 2018; Tordoff *et al*., 2021), this review focuses on *E. coli*.

## Engineered circuits for spatial patterning

**Fig. 1:**
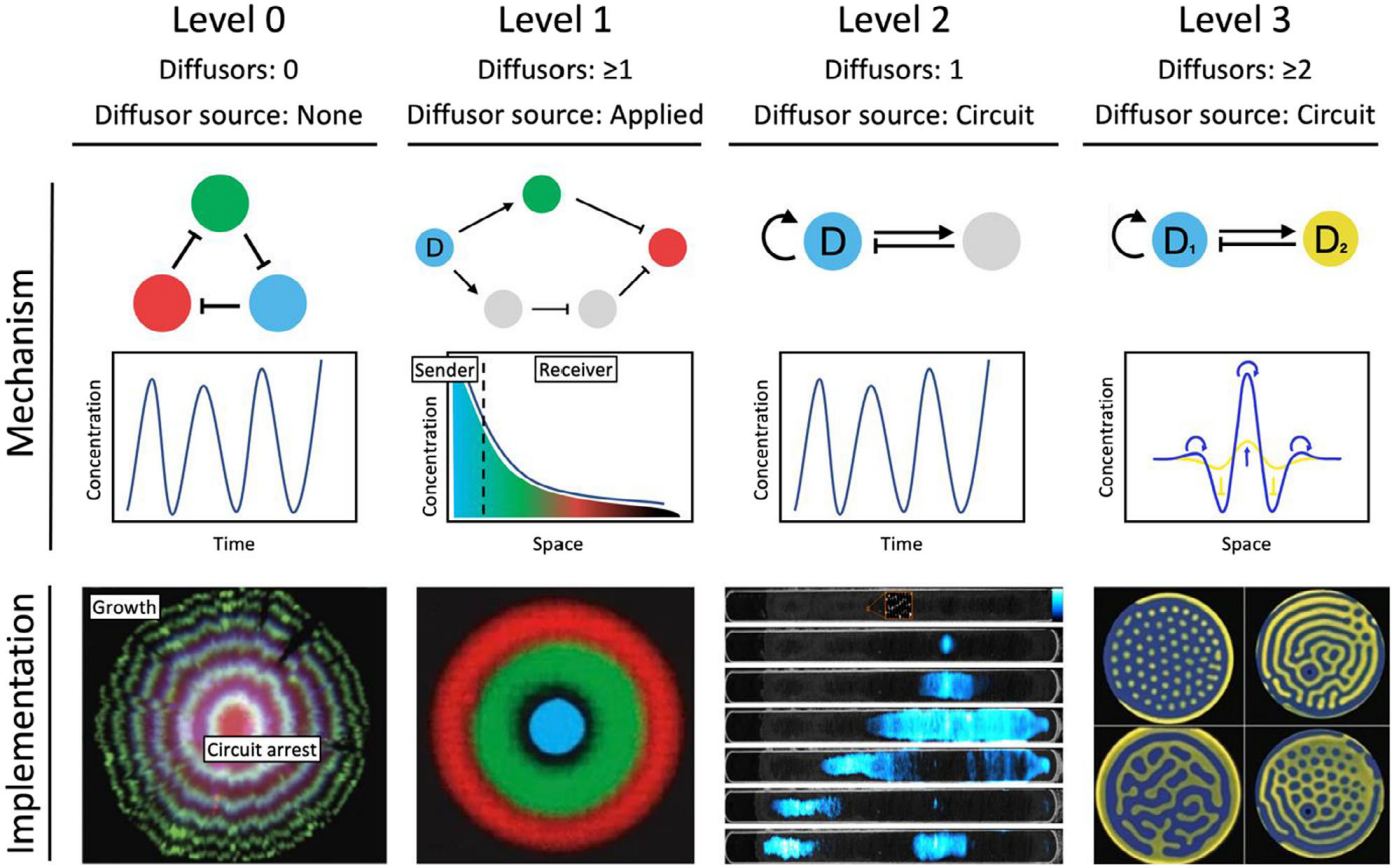
Four levels of regulatory complexity in engineered spatial patterning systems. Each level is divided into an example circuit, and the resulting pattern upon implementation. Diffusing components of the circuit are labelled with a “D”, non‐diffusing nodes are unlabelled. The colour of each node corresponds to the colour of the reporter in the respective implementation. Level 0: synchronized repressilator circuit implemented in a growing bacterial colony (Potvin‐Trottier *et al*., 2016). The plot shows the circuit oscillations in single cells or stirred liquid culture. Level 1: incoherent feedforward circuit, where the diffusor‐producing sender cells (cyan) are placed in the middle of a bacterial lawn (Basu *et al*., 2005). The plot shows the concentration gradient of the diffusor away from the centre of the lawn. Level 2: self‐activation and feedback inhibition circuit with one dynamically regulated diffusor creates spatial propagating waves and spatially synchronized oscillations (not shown) (Danino *et al*., 2010). The plot shows the oscillations of the circuit in single cells, or in a cell population. Level 3: self‐activation and lateral‐inhibition circuit with two dynamically regulated diffusors creates stationary Turing patterns in the TuIS chemical system (Horváth *et al*., 2009). The plot shows the localized, self‐activating positive feedback of the slow‐diffusing species D_1_ (blue curve) and the lateral inhibition of the fast‐diffusing species D_2_ (yellow curve).

To consider the problem of synthetic patterning systems systematically, here we suggest grouping them into four levels according to their design characteristics (Fig. 1). Level 0 circuits do not contain any synthetic signals that diffuse through normal Fickian diffusion; instead, spatial structure emerges by other processes, such as cellular growth. Level 1 systems rely on one or more diffusing components whose production is not dynamically regulated by the circuit. Level 2 systems incorporate a single diffusible component, which is dynamically regulated by the circuit components. Level 3 systems use multiple dynamically regulated diffusible components.

Spatial systems where the diffusing components are not dynamically regulated by the circuit (Level 1) were the first to be engineered in *E. coli* (Basu *et al*., 2005). Stripe patterning systems are the most prominent example among Level 1 circuits. Initially, diffusor gradients were interpreted by intracellular incoherent feedforward circuits, to form rings of gene expression at intermediate distances from their source (Basu *et al*., 2005; Schaerli *et al*., 2014; Kong *et al*., 2017). Bistable mutually inhibitory circuits were also used to interpret morphogen gradients, forming systems that can robustly generate sharp boundaries between two or more spatial regions of gene expression (Barbier *et al*., 2020; Grant *et al*., 2020).

Hierarchical patterning is a scalable Level 1 system, implemented in a recently engineered circuit in *E. coli* (Boehm *et al*., 2018). Two diffusor sources at the edges of a spatial domain are interpreted by an AND gate circuit to lead to three distinct spatial partitions. In theory, additional diffusing species and AND gates could be introduced to generate increasingly complex structures: for example (2*n*‐1) spatial domains could be generated from *n* orthogonal diffusing signals in a one‐dimensional space. While this type of patterning can explain some developmental patterning programs, such as that of the vertebrate neural tube (Briscoe and Small, 2015), it fails to capture self‐organizing periodic structures, such as digit patterning in the chick limb bud (Sheth *et al*., 2012; Raspopovic *et al*., 2014).

Spatial patterns are often shaped by the complex interaction of the circuit components with cellular growth and other biological and physical properties of the system. This interplay is highlighted in some recently engineered Level 0 systems, where a synchronized circuit of the repressilator (Elowitz and Leibler, 2000) was used to produce periodic concentric ring patterns in growing colonies of cells, in the absence of diffusing signals (Potvin‐Trottier *et al*., 2016; Riglar *et al*., 2019). The mechanism that provides spatial structure consists of a combination of growth at the edge of the bacterial colony, and of the arrest of circuit activity at the colony interior.

Reaction–diffusion systems, where the diffusor is dynamically regulated by the circuit components (Level 2), have also been engineered successfully (Danino *et al*., 2010; Payne *et al*., 2013). Unlike Level 1, Level 2 systems generally do not rely on pre‐patterns or positional information. For this group, engineering was mainly focused on circuits with a self‐activating and a laterally inhibiting component. A prominent example is the oscillator with a diffusible positive feedback, observed to generate spatially synchronized oscillations and propagating waves (Danino *et al*., 2010). A further example with a diffusible inhibiting component, but lacking a diffusible positive feedback, was shown to produce a ring pattern in growing colonies of cells, which is not reliant on diffusor gradients (Payne *et al*., 2013; Cao *et al*., 2016). All the systems mentioned above rely on a single diffusive component; the engineering of these systems becomes increasingly challenging for more diffusing species.

While numerous successes were achieved with Level 1 and 2 spatial circuits, successfully engineering Level 3 systems, consisting of multiple dynamically regulated diffusors, is still in its infancy. Turing patterns are the most prominent example of Level 3 systems; they are formed by reaction–diffusion circuits of at least two diffusors, where generally the first is self‐activating, whereas the second performs a lateral inhibition (Turing, 1952; Gierer and Meinhardt, 1972; Scholes *et al*., 2019). Classical, deterministic Turing patterns self‐organize into periodic spot, stripe or labyrinthine spatial structures (Horváth *et al*., 2009; Asakura *et al*., 2011; Murray, 2013). Originally, they were formulated mathematically with little regard to biological context (Turing, 1952). Computationally, many biological candidate networks were found to produce Turing patterns (Marcon *et al*., 2016; Zheng *et al*., 2016; Scholes *et al*., 2019). However, engineering them remains difficult mainly because of their high sensitivity to changes in system parameters (Maini *et al*., 2012; Scholes *et al*., 2019). The issue of fine‐tuning is exacerbated by the lack of appropriately tuneable components to achieve the narrow parameter space in which classical Turing patterns occur.

Greater success was seen with stochastic Turing patterns because their fine‐tuning requirements are more relaxed (Butler and Goldenfeld, 2011). Stochastic Turing patterns were recently engineered in *E. coli* with a circuit implemented according to the self‐activation and lateral inhibition topology, with two diffusible quorum‐sensing signals (Karig *et al*., 2018). While easier to engineer, stochastic Turing patterns display more irregularity in their periodic spatial structure (Butler and Goldenfeld, 2011; Karig *et al*., 2018). Solitary structures are another possible mechanism for periodic patterning due to their close resemblance to some natural patterns (Sekine *et al*., 2018). They can also be formed by activator–inhibitor reaction–diffusion systems; however, their fine‐tuning requirements are more relaxed compared to Turing patterns, and might therefore be easier to build (Koga and Kuramoto, 1980; Purwins *et al*., 2010). Even though they are still an unsolved engineering problem, solitary patterns were recently observed in a refactored Nodal–Lefty system in HEK cells (Sekine *et al*., 2018).

While elusive in synthetic biology, regular‐repeat Turing patterns were more readily observed in chemical reaction systems, where they were first detected in the early 1990s in the chlorite−iodide−malonic acid (CIMA) reaction (Castets *et al*., 1990; Lengyel *et al*., 1993). Turing patterns were then also discovered in the thiourea–iodate–sulfite (TuIS) reaction with a rational design approach (Horváth *et al*., 2009). Unlike biological systems, chemical reactions are reliably described by the simpler laws of mass action, and system parameters can often be identified (Turányi, 1994; Kügler *et al*., 2009; Pušnik *et al*., 2019; Yeoh *et al*., 2019; Tica *et al*., 2020). Furthermore, the tuning of these systems by changing initial reactant concentration or temperature is easily achieved and has predictable effects on the dynamics of the system (Horváth *et al*., 2009; Carballido‐Landeira *et al*., 2010; Asakura *et al*., 2011). Lastly, the systems are easily isolated from external interacting components; this is difficult to achieve with biological systems where cross‐talk between synthetic parts and with the cellular chassis is inevitable (Ceroni *et al*., 2015; Nielsen *et al*., 2016; Butzin and Mather, 2018; Müller *et al*., 2019; Du *et al*., 2020). These and other related factors made chemical reaction systems suited to support such a fine‐tuned phenomenon as Turing patterns. However, recent advances with the parametrization of synthetic genetic circuits may open new possibilities also in the field of synthetic biology (Espah Borujeni *et al*., 2020).

Multi‐diffusor systems were historically held back by the lack of a diverse palette of well‐characterized diffusible components. Studies tried to circumvent this shortage, for example, by considering the *E. coli* cell chassis to be one of the diffusors (Duran‐Nebreda *et al*., 2021). However, cell growth within a bacterial colony differs significantly from classical diffusion because it is not directionally unbiased. The movement of cells in space is limited within a colony and mainly happens outwards, in the direction of growth. Due to developments in directed evolution, genome mining and metabolic engineering, more well‐characterized diffusible components have recently become available (Meyer *et al*., 2019; Du *et al*., 2020). We expect these advances to be pivotal in the further development of spatial patterning systems, particularly of multi‐diffusor circuits.

The same diffusible synthetic components were also used in the engineering of spatially distributed computing systems, where neighbouring bacterial colonies containing simple logic gate circuits communicate by secreting diffusible signals (Tamsir *et al*., 2011; Du *et al*., 2020). Spatially distributed systems enable complex biological computation ranging from basic logic operations (Du *et al*., 2020) to more complex neural‐like computing (Li *et al*., 2021). These systems do not fall in any of the circuit categories introduced in this study due to their spatially distributed nature. Even though outside the scope of this article, these types of systems would also directly benefit from the development of novel well‐characterized signalling modules.

## Novel diffusible components

Historically, the biggest hindrance to the development of spatial systems with two or more dynamically regulated diffusors is the lack of well‐characterized, robust and tuneable diffusing components for *E. coli* (Scholes and Isalan, 2017). The basic criteria that synthetic signalling modules need to satisfy are: (i) diffusion and bidirectional passage across cellular boundaries; (ii) ability to regulate gene expression; (iii) simple synthesis pathways in *E. coli*, to avoid metabolic burden and issues with refactoring overly complex systems; (iv) orthogonality to other synthetic components and endogenous *E. coli* chemistry; (v) it is also desirable that the signals are well‐characterized and optimized for model‐based rational engineering.

Among potential diffusible components, quorum‐sensing homoserine lactones (HSLs) are most widely used in *E. coli* synthetic biology (Basu *et al*., 2005; Danino *et al*., 2010; Karig *et al*., 2018). HSLs are well‐studied and were recently reviewed in the context of synthetic biology and pattern engineering (Papenfort and Bassler, 2016; Boo *et al*., 2021). While being versatile and easy to implement, they also possess limitations, which mainly stem from their similar chemistry. First, even though orthogonal HSLs exist, cross‐talk between them is common (Boedicker and Nealson, 2016; Silva *et al*., 2017; Tekel *et al*., 2019; Du *et al*., 2020). In addition, engineering differential diffusion with pairs of HSLs can be challenging; this is of particular interest for Turing pattern engineering and could also be of interest with other spatiotemporal systems where space scale separation is needed (Lengyel and Epstein, 1992; Szalai and De Kepper, 2008; Horváth *et al*., 2009). While quorum sensing is a highly effective solution to implement cell–cell communication in prokaryotes, this article aims to move beyond it and focus on novel non‐quorum‐sensing signals.

Recently, 12 different small molecule inducible genetic systems were optimized for use in *E. coli* synthetic biology (Meyer *et al*., 2019). These were incorporated in the 'Marionette' strain, which provides the capability of regulating 12 genes simultaneously and independently. However, to use these inducible systems in a Level 2 or 3 spatial circuit, the small molecules must be produced endogenously from freely available precursors. Among the Marionette components, at least six could potentially be easily produced by *E. coli* with enzymes ported from other microorganisms: excluding quorum‐sensing systems, these are DAPG, salicylate, protocatechuate, naringenin, vanillate, acrylate).

These avenues were further explored in a recent study where six novel, orthogonal, small‐molecule inducers were developed for use in *E. coli* synthetic biology (Du *et al*., 2020). Both their inducible genetic components and synthesis mechanisms were developed and optimized for synthetic cell–cell communication. A screen of the literature shows that many more diffusible signals could be ported to *E. coli*, as candidates for well‐behaving signalling modules. Table 1 provides a list of the recently discovered signals and of the potential candidates. Although this review focuses on *E. coli,* some studies indicate that these diffusors may be ported to other prokaryotes as well as some eukaryotes for a wider range of applications. For instance, three of the molecules in Table 1 have successfully been engineered in *E. coli, S. cerevisiae* and mammalian cells (HEK‐293T) (Du *et al*., 2020).

**Table 1:** Novel diffusible signals for *E. coli* synthetic biology.

Component	Pubchem ID	Synthesis mechanism	Degradation	Receptor	Max fold induction	Molecular weight (Da)	Diffusion rate (mm^2^ h^−1^)	References
*Novel diffusible signals optimized for synthetic biology*								
DAPG	16547	*phlACBD*	*phlG*	*phlF*	1380	210.18	2.66	Bottiglieri and Keel (2006), Meyer *et al*. (2019), Du *et al*. (2020)
Salicylate	338	*pchBA/irp9/ybts*	*nahG*	*nahR*	47	138.121	3.22	Du *et al*. (2020)
p‐Coumaroyl‐HSL	71311837	*rpaI, 4cI, tal*	*aiiA*	*rpaR*	170	247.25	2.47	Liao *et al*. (2018), Du *et al*. (2020)
Isovaleryl‐HSL	71627311	*bjal, bkdFGH, ipdA1*	*aiiA*	*bjaR*	350	185.22	2.82	Liao *et al*. (2018), Du *et al*. (2020)
MMF	N/A	*mmfLHP*	*N/A*	*mmfR*	26	198.22	2.73	Du *et al*. (2020)
Naringenin	932	*chs, chi, 4cl, tal*	*fns*	*fdeR*	16	272.25	2.37	Lee *et al*. (2015), Du *et al*. (2020)
C_4_‐HSL	10130163	*rhlI*	*aiiA*	*rhlR*	124	171.2	2.92	Du *et al*. (2020)
3OC_6_‐HSL	119133	*luxI*	*aiiA*	*luxR*	185	213.23	2.64	Du *et al*. (2020)
C_8_‐HSL	6914579	*cepI*	*aiiA*	*cepR*	150	227.3	2.57	Du *et al*. (2020)
3OC_12_‐HSL	3246941	*lasI*	*aiiA*	*lasR*	82	297.194	2.28	Du *et al*. (2020)
Cumate	10820	*N/A*	*N/A*	*cymR*	860	164.2	2.97	Meyer *et al*. (2019)
Vanillate	8468	*tal, c3h, comt, fcs, ech, ligV*	*ligM*	*vanR*	1250	168.15	2.94	Ni *et al*. (2015), Wu *et al*. (2018), Meyer *et al*. (2019)
IPTG	656894	*N/A*	*N/A*	*lacI*	688	238.3	2.51	Meyer *et al*. (2019)
ATC	54675758	*N/A*	*N/A*	*tetR*	490	426.4	1.95	Meyer *et al*. (2019)
l‐arabinose	439195	*N/A*	*N/A*	*araC/araE*	500	150.13	3.10	Meyer *et al*. (2019)
Choline	6209	*N/A*	*N/A*	*betI*	306	139.62	3.20	Meyer *et al*. (2019)
Protocatechuate	19	*aroZ / pobA*	*aroY*	*pcaU*	356	154.12	3.06	Martin *et al*. (2013), Wang *et al*. (2017a), Meyer *et al*. (2019)
3OHC_14_‐HSL	11681427	*cinI*	*aiiA*	*cinR*	500	327.46	2.19	Meyer *et al*. (2019)
Acrylate	6581	*aspA, panD, act, acl2, yciA*	*N/A*	*acuR*	84	72.06	4.39	Meyer *et al*. (2019), Ko *et al*. (2020)
Erythromycin	12560	*N/A*	*N/A*	*mphR,ery*	37	733.9	1.56	Zhang *et al*. (2010), Meyer *et al*. (2019)
*Potential diffusible signals for synthetic biology*								
Kynurenine	846	*kynAB*	*kynU*	*kynR*		208.21	2.67	Kurnasov *et al*. (2003), Hanko *et al*. (2020)
Itaconate	811	*cadA*	*ripABC*	*itcR*	215	130.1	3.31	Okamoto *et al*. (2014), Hanko *et al*. (2018, 2020), Barbier *et al*. (2020)
Acetoin	179	*budAB*	*pc‐acoABCL*	*acoR*		88.11	4.00	Huang *et al*. (1999), Ali *et al*. (2001), Delamarre and Batt (2006), Silbersack *et al*. (2006), Vivijs *et al*. (2014), Hanko *et al*. (2020)
Trigonelline	5570	*ctgS1/ctgS2*	*tgnAB*	*nodD*		137.14	3.23	Schmidt *et al*. (1986), Ashihara (2008), Mizuno *et al*. (2014), Wang *et al*. (2017b), Perchat *et al*. (2018)
Benzoate	242	*pal, 4cl, phdBCE*	*benABCD*	*benM*	3700	121.11	3.42	Neidle *et al*. (1987), Otto *et al*. (2020)
*cis,cis*‐Muconate	5280518	*pobA, aroY, catA*	*catBC*	*catR/benM*		142.11	3.18	(Parsek *et al*., 1992, Sengupta *et al*., 2015, Skjoedt *et al*., 2016, Choi *et al*., 2020)
Luteolin	5280445	*tal, 4cl, chs, chi, fns, f3h*	*spnK*	*nodD*		286.24	2.32	Schmidt *et al*. (1986), Suominen *et al*. (2003), Peck *et al*. (2006), Marín *et al*. (2017), Bashyal *et al*. (2019), De Paepe *et al*. (2019)
Apigenin	5280443	*tal, 4cl, chs, fns*	*pomt7 / f3h*	*nodD*		270.24	2.38	Lee *et al*. (2015), Marín *et al*. (2017), De Paepe *et al*. (2019)
Kaempferol	5280863	*tal, 4cl, chs, chi, f3h, fls*	*N/A*	*qdoR*		286.24	2.32	Siedler *et al*. (2014), Stahlhut *et al*. (2015)
Quercetin	5280343	*tal, 4cl, chs, chi, f3h, fls, fmo*	*yhhW*	*qdoR*		302.23	2.26	Adams and Jia (2005), Stahlhut *et al*. (2015), An *et al*. (2016), Marín *et al*. (2018)
Ectoine	126041	*ectABC*	*ectD*	*ehuB*		142.16	3.18	(Jebbar *et al*., 2005, He *et al*., 2015, Richter *et al*., 2019)
Nicotinate	938	*pncA*	*nnmt*	*nicS*		123.11	3.39	(Joshi and Handler, 1960, Shats *et al*., 2020)
Phloretin	4788	*tal, er, 4cl, chs*	*phy*	*pmeR*		274.26	2.36	Schoefer *et al*. (2004), Vargas *et al*. (2011), Liu *et al*. (2022)
Phenylglyoxylate	1548898	*dmdh*	*mdlC*	*phgR*		150.13	3.10	Gunsalus *et al*. (1953), Tang *et al*. (2018), Hanko *et al*. (2020)

The table shows some recently optimized diffusible signals collected from Meyer *et al*. (2019) and Du *et al*. (2020), and potential diffusible signals that were not yet optimized for synthetic gene circuit engineering. Synthesis and degradation pathways are suggested for each of the molecules where available. The transcription factors regulated by each of the molecules are also shown; some basic parameters of their genetic response systems are shown where available. The molecular weights are used to predict their diffusion coefficients in D_2_O (Evans *et al*., 2018). DAPG, diacetylphloroglucinol; MMF, methylenomycin furan; N/A, not available to date.

For the successful implementation of these signals, it is important to optimize both the synthetic and the sensing components, where the efficiency in the endogenous synthetic system should meet the sensitivity of the sensing component. For example, it could easily happen that the endogenous synthetic mechanisms do not produce enough inducer to fully activate the sensors, leading to a poor dynamic range in their response. For this purpose, endogenous metabolic pathways may need to be tuned to increase precursor availability (Ni *et al*., 2015) or to avoid diffusor degradation (Adams and Jia, 2005). The development of robust diffusible signals and of bacterial strains that can reliably support this signalling is pivotal for the field of spatial pattern engineering and will potentially benefit synthetic biology in general.

## Conclusion

The engineering of biological patterns could help untangle the complex mechanisms of development (Davies, 2017) and revolutionize organoid engineering and materials science. While many interesting patterns have already been built, the potential for innovation is still great. This is particularly true for multi‐diffusor circuits, which could potentially show more diverse and complex spatiotemporal behaviours (Boehm *et al*., 2018; Barbier *et al*., 2020; Grant *et al*., 2020). We argue that the recent development of novel small‐molecule diffusible signals will contribute to a development of spatial circuits, particularly of those with multiple diffusible components. We anticipate that recently discovered diffusive signals will enable synthetic biologists to engineer increasingly intricate, robust and tuneable spatial structures.

## Conflict of interest

None declared.
